# Curcumin inhibits the proliferation and migration of vascular smooth muscle cells by targeting the chemerin / CMKLR1 / LCN2 axis

**DOI:** 10.18632/aging.202980

**Published:** 2021-05-24

**Authors:** Yaqiong He, Rongning Wang, Peng Zhang, Jianlong Yan, Nan Gong, Yuhang Li, Shaohong Dong

**Affiliations:** 1 Department of Cardiology, Shenzhen People’s Hospital, Jinan University, Shenzhen 518000, Guangdong, China; 2 Department of Orthopedics, Third Affiliated Hospital of Sun Yat-Sen University, Guangzhou 510630, Guangdong, China

**Keywords:** atherosclerosis, CMKLR1, LCN2, curcumin, VSMC

## Abstract

Atherosclerosis (AS) is a chronic progressive inflammatory disease and a leading cause of death worldwide. Being a novel adipokine, chemerin is reported to be positively correlated with the severity of AS, yet its underlying mechanisms in AS remains elusive. It is well-known that AS development is significantly attributed to abnormal proliferation and migration of vascular smooth muscle cells (VSMCs). Therefore, we investigated the role of the chemerin / chemokine-like receptor 1 (CMKLR1, chemerin receptor) signaling, and the potential therapeutic effect of curcumin in VSMCs proliferation and migration during AS by establishing a high fat diet (HFD) mouse model. We found that CMKLR1 was highly expressed in HFD-induced AS tissues and that its expression level was positively correlated with aortic proliferation. Knockdown of CMKLR1 significantly inhibited VSMCs proliferation and migration, as evidenced by the EdU-incorporation assay, wound healing assay, and the induction of proliferating cell nuclear antigen (PCNA) and matrix metalloproteinase-9 (MMP-9) expression. Furthermore, we discovered that Lipocalin-2 (LCN2) acts as a key factor involved in CMKLR1-mediated VSMCs proliferation and migration via the p38 / MAPK and Wnt / β-catenin signaling pathways, and we demonstrated that curcumin inhibits VSMCs proliferation and migration by inhibiting chemerin / CMKLR1 / LCN2, thereby reducing AS progression. Our findings suggest that chemerin / CMKLR1 activation promotes the development of AS; hence, targeting the chemerin / CMKLR1 / LCN2 signaling pathway may be a reasonable treatment modality for AS.

## INTRODUCTION

Atherosclerosis (AS) is a chronic progressive inflammatory disease and a leading cause of death worldwide [1, 2]. Clinically, atherosclerosis remains a major public health challenge for long-term healthy living. Additionally, abnormal proliferation and migration of vascular smooth muscle cells (VSMCs) are key events contribute to development of AS [3–5]. Recently, several adipokines, such as adiponectin and visfatin, have been demonstrated to play a crucial role in the abnormal proliferation and migration of VSMCs, and targeting these adipokines has inhibited the development of AS [6, 7]. These findings suggest that intensive study of adipokines in VSMCs may provide a new clue for the treatment of AS.

chemerin, which is also called retinoic acid receptor response protein 2 (RARRES2) or tazarotene-induced gene 2 protein (Tig2), is a novel adipokine [8]. Clinical studies have confirmed that circulating chemerin levels are positively correlated with the severity of AS [9]. More importantly, we have previously demonstrated that chemerin stimulates AS progression in apo-lipoprotein E^−/−^ (ApoE^−/−^) mice [10]. These results suggested that chemerin signaling may be crucial to AS development. Chemokine-like receptor 1 (CMKLR1, also known as ChemR23), the first confirmed chemerin receptor, is highly expressed in plasmacytoid dendritic cells, macrophages, adipocytes, and endothelial cells [11]. The chemerin / CMKLR1 axis is a complex network involved in the regulation of immune responses, intimal hyperplasia, hypertension, and myocardial infarction [12–14]. Recent studies have revealed the role of chemerin / CMKLR1 in the cellular proliferation and migration of cancers [15]. However, the role of chemerin / CMKLR1 in atherosclerosis is still a highly contentious issue [10, 16]. Therefore, determining the functional and mechanistic regulation of chemerin / CMKLR1 in VSMCs is of interest.

Natural products (NPs), as the basis for many widely used drugs, have been increasingly recognized. Curcumin, which is a component of turmeric, has been shown to have excellent therapeutic effects in a variety of aging-related diseases, such as tumors, neurodegenerative diseases, diabetes, and chronic inflammation [17–20]. The latest research confirms that long-term curcumin treatment can reduce plasma and liver cholesterol, thereby inhibiting early atherosclerotic lesions [21]. However, the role and mechanism of curcumin in the regulation of chemerin / CMKLR1 signaling remains unclear.

In this study, we investigated the role of the chemerin / CMKLR1 signaling and the potential therapeutic effect of curcumin in VSMCs proliferation and migration during AS by establishing a high fat diet (HFD) mouse model.

## RESULTS

### Chemerin / CMKLR1 is highly expressed in HFD-induced AS and that its expression level is positively correlated with cellular proliferation

**Figure 1:**
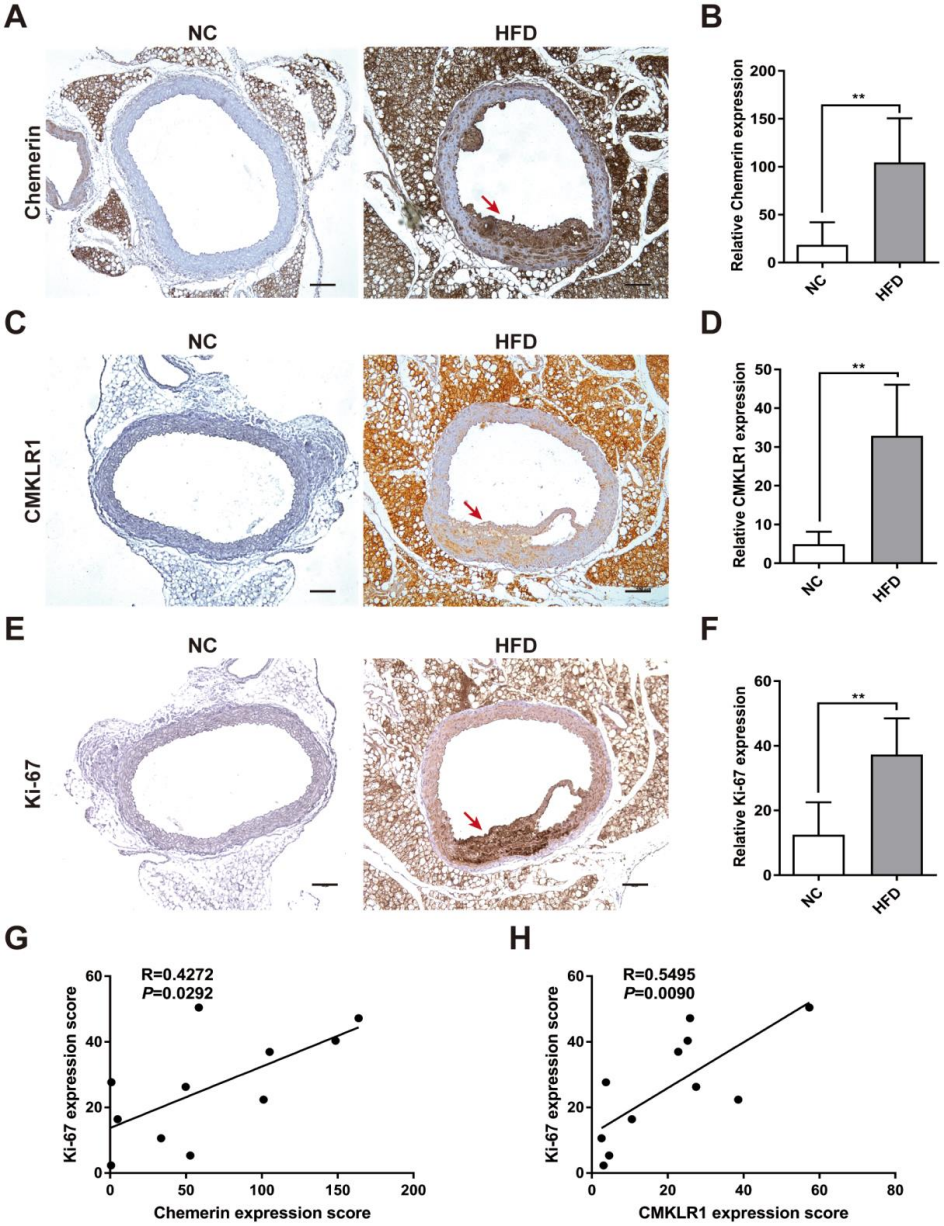
**Chemerin / CMKLR1 is overexpressed in the aorta tissues of HFD ApoE-/- mice.** A high fat diet (HFD) apo-lipoprotein E−/− (ApoE-/-) mouse model was established to determine the expression of the chemerin / chemokine-like receptor 1 (CMKLR1) axis in atherosclerotic aorta tissues. (**A**) Immunohistochemical (IHC) analysis of chemerin expression in aorta tissues of negative control (NC, n = 5) and HFD (n = 6) mice. Scale bar = 100 μm. Red arrows indicate plaques. (**B**) The chemerin expression score analysis data are presented as means ± standard deviations (SDs). **, *p*< 0.01. (**C**) Representative images of CMKLR1 expression in aorta tissues following IHC analysis. Scale bar = 100 μm. Red arrows indicate plaques. (**D**) CMKLR1 expression score analysis. **, *p*< 0.01. (**E**, **F**) IHC analysis of Ki-67 expression in the aorta tissues of normal and HFD mice. Scale bar = 100 μm. Red arrows indicate plaques. Data from Ki-67 expression score analysis are presented as means ± SDs. **, *p*< 0.01. (**G**, **H**) The correlations of chemerin / CMKLR1 and Ki-67 were calculated using the Spearman method.

chemerin has previously been shown to be correlated with AS, yet its underlying mechanisms remains elusive [9]. In this study, an HFD-induced AS mouse model was established, which exhibited significant extensive aortic atherosclerotic lesions (Figure 1A). We first analyzed arterial levels of chemerin / CMKLR1 in HFD-induced AS mice via immunohistochemistry (IHC) assay. As shown in Figure 1A–1D, aortic expressions of chemerin / CMKLR1 increased significantly in HFD mice. It's worth noting that a high expression level of Chemerin, but not CMKLR1, was observed in perivascular adipose tissue without HFD induction (Figure 1A, 1C). Given that Ki-67 is one of the most widely used markers for assessing proliferation [22], we then sought to correlate the expression of chemerin / CMKLR1 with Ki-67 in arterial tissues. As shown in Figure 1E, 1F, Ki-67 expression increased remarkably both in aorta and perivascular adipose tissue. Chemerin and CMKLR1 expressions were positively correlated with the Ki-67 level in the aorta (Figure 1G, 1H). These results suggest that high chemerin / CMKLR1 expression may play a role in cellular proliferation and the development of AS.

### Knockdown of CMKLR1 inhibits VSMCs proliferation and migration

**Figure 2:**
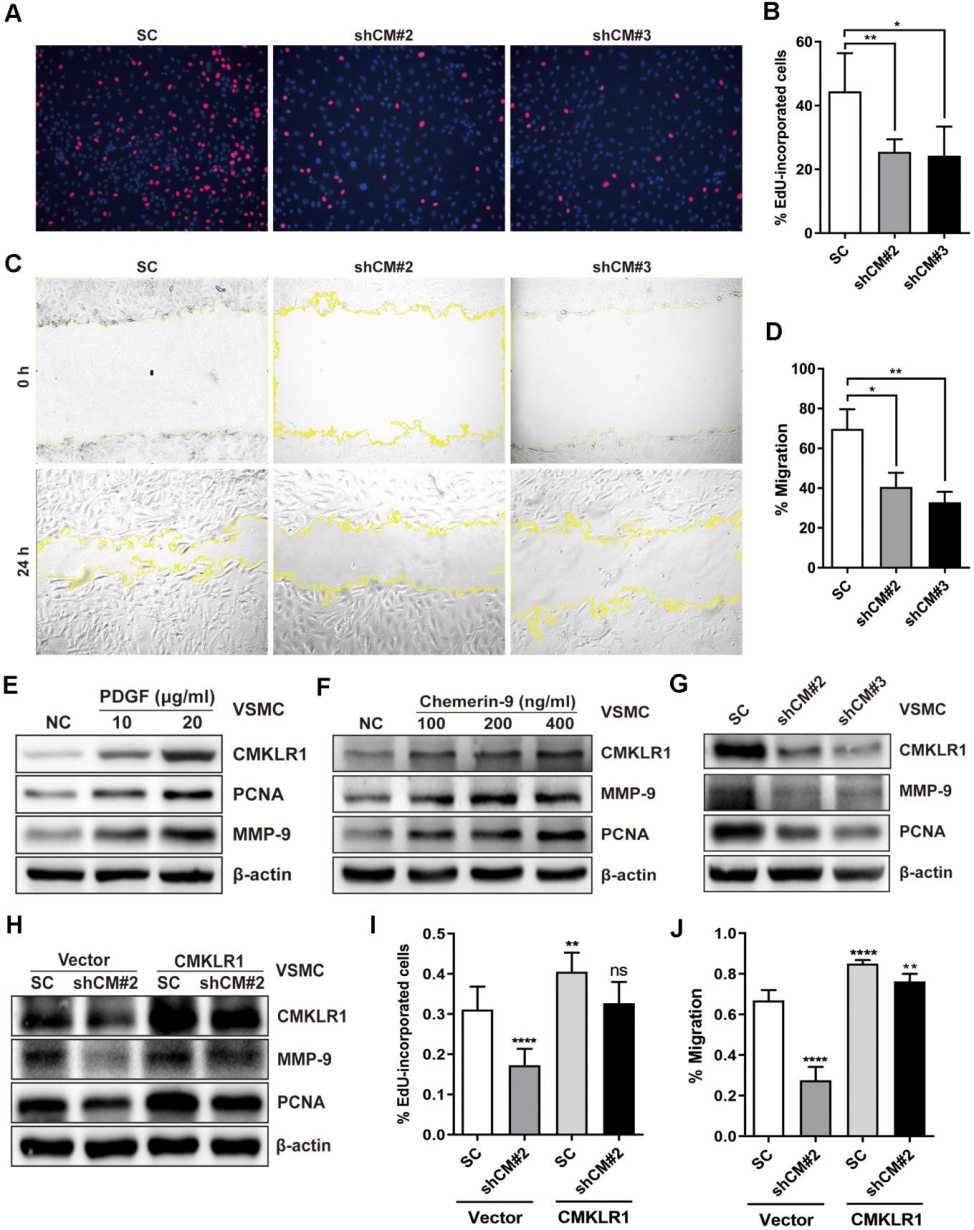
**Downregulation of CMKLR1 inhibit VSMCs proliferation and migration.** (**A**, **B**) Percentage of 5-Ethynyl-2'-deoxyuridine (EdU)-incorporated cells following the application of two independent shRNAs targeting CMKLR1 (scramble control, SC; shCMKLR1#2, shCM#2; shCMKLR1#3, shCM#3) to knock down CMKLR1 expression in mice vascular smooth muscle cells (VSMCs). *, *p*< 0.05; **, *p*< 0.01. (**C**, **D**) Percentage of cell migration as determined by wound healing assay. *, *p*< 0.05; **, *p*< 0.01. (**E**) Western blots showing CMKLR1, MMP-9 and PCNA expressions in cells treated with PDGF (48 h) for different durations. (**F**) Western blots showing CMKLR1, matrix metalloproteinase-9 (MMP-9) and proliferating cell nuclear antigen (PCNA) expression in cells treated with chemerin-9 (48 h) for different durations. (**G**) Western blots showing CMKLR1, MMP-9 and PCNA expressions with or without CMKLR1 knockdown. (**H**) Western blots showing CMKLR1, MMP-9 and PCNA expressions following the application of plasmids encoding CMKLR1 to overexpress CMKLR1 expression in VSMCs with or without CMKLR1 knockdown. (**I**) Analysis of the percentage EdU-incorporated cells with or without LCN2 depletion. ****, *p*< 0.0001; **, *p*< 0.01; ns, no significant difference. (**J**) Percentage of cell migration as determined by wound healing assay. ****, *p*< 0.0001; **, *p*< 0.01.

Next, we investigated the possible effects of CMKLR1 on the proliferation and migration of VSMCs *in vitro*. CMKLR1 knockdown significantly inhibited VSMCs proliferation, as evidenced by the 5-Ethynyl-2'-deoxyuridine (EdU) incorporation assay results (Figure 2A, 2B). We then demonstrated via the wound healing assay that CMKLR1 knockdown remarkably inhibited VSMCs migration (Figure 2C, 2D). We then confirmed that CMKLR1 depletion induced cell cycle G0/G1 arrest which was associated with the inhibition of cellular proliferation and migration (Supplementary Figure 1A, 1B). Interestingly, knock down of CMKLR1 also triggered VSMCs apoptosis, which may increase plaque fragility and inflammation in AS (Supplementary Figure 1C, 1D). PDGF is one of the most common stimulants for VSMCs proliferation and migration *in vitro*; and we demonstrated that the expression of CMKLR1 was markedly upregulated after treatment of PDGF in a concentration dependent manner (Figure 2E). Chemerin-9, a special CMKLR1 agonist [23], was used to confirm the role of CMKLR1 in VSMCs proliferation and migration, and we found that chemerin-9 treatment significantly increased the protein expressions of both proliferating cell nuclear antigen (PCNA) [24], a widely used marker of proliferation, and matrix metalloproteinase-9 (MMP-9), which has been demonstrated to be related with cellular migration [25] (Figure 2F). CMKLR1 knockdown consistently inhibited the protein expressions of PCNA and MMP-9 (Figure 2G). In contrast, overexpression of CMKLR1 by transiently transfection of the plasmid encoding CMKLR1 markedly recovered the expressions of PCNA and MMP-9 as well as VSMCs proliferation and migration inhibited by CMKLR1 depletion, indicating the specific role of CMKLR1 in upregulating cellular proliferation and migration (Figure 2H–2J). Taken together, these results demonstrate the crucial role of CMKLR1 in upregulating PCNA and MMP-9 expressions and thus the VSMCs proliferation and migration.

### LCN2 plays a role in inhibition of VSMCs proliferation and migration induced by CMKLR1 knockdown

**Figure 3:**
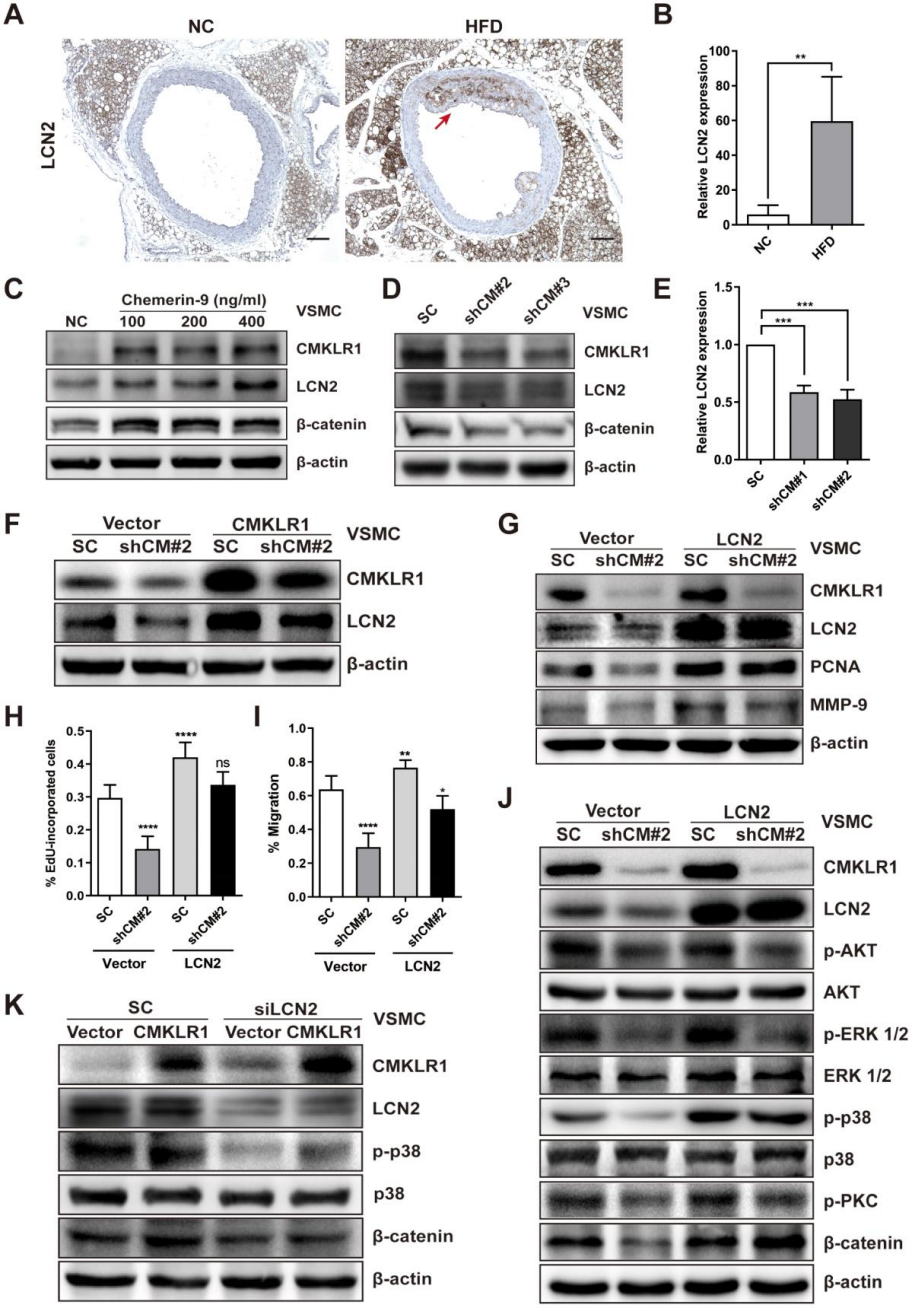
**LCN2 is essential for the inhibition of VSMCs proliferation by CMKLR1 depletion.** (**A**) IHC analysis of Lipocalin-2 (LCN2) expression in aorta tissues of mice fed with normal and HFD. Scale bar = 100 μm. Red arrows indicate plaques. (**B**) Chemerin expression score analysis data presented as the means ± SDs. **, *p*< 0.01. (**C**) Western blots showing LCN2, MMP-9, and β-catenin expression in cells treated with chemerin-9 (48 h) for different durations. (**D**) Western blot showing LCN2, MMP-9, and β-catenin expression, with or without CMKLR1 knockdown. (**E**) mRNA expression of LCN2, with or without CMKLR1 depletion, as determined by quantitative real-time PCR. (**F**) Western blots showing CMKLR1 and LCN2 expression following the application of plasmids encoding CMKLR1 to overexpress CMKLR1 expression in VSMCs with or without CMKLR1 depletion. (**G**) Western blots showing CMKLR1, LCN2, PCNA and MMP-9 expression following the application of plasmids encoding LCN2 to overexpress LCN2 expression in VSMCs with or without CMKLR1 depletion. (**H**) Analysis of the percentage EdU-incorporated cells. ****, *p*< 0.0001; ns, no significant difference. (**I**) Cell migration as determined by wound healing assay. Percentage of cell migration data presented as means ± SDs. ****, *p*< 0.0001; **, *p*< 0.01; *, *p*< 0.05. (**J**) Western blots showing CMKLR1, LCN2, p-AKT, AKT, p-ERK 1/2, ERK 1/2, p-p38, p38, p-PKC and β-catenin expression following the application of plasmids encoding LCN2 to overexpress LCN2 expression in VSMCs with or without CMKLR1 depletion. (**K**) Western blots showing CMKLR1, LCN2, p-p38, p38 and β-catenin expression following the transfection of small interfering RNA (siRNA) targeting LCN2 (siLCN2) into VSMCs with or without CMKLR1 overexpression.

Then, we sought to identify the key factor responding to CMKLR1 knockdown-induced inhibition of VSMCs proliferation and migration. Recently, Lipocalin-2 (LCN2) was found to be a key regulator of cellular proliferation via Wnt / β-catenin signaling [26], and a previous study demonstrated that serum chemerin and LCN2 levels were positively correlated in patients suffered from metabolic and inflammation-related diseases [27]; however, the relationship between CMKLR1 and LCN2 remains unknown. Thus, we first examined LCN2 expression in HFD-induced AS mice via IHC assay and found that aortic LCN2 expression increased significantly in HFD mice, and was positively correlated with CMKLR1 expression (Figure 3A, 3B). We further confirmed that Chemerin-9 treatment induced the LCN2 expression (Figure 3C). CMKLR1 knockdown consistently inhibited the protein and mRNA expression of LCN2 (Figure 3D, 3E). In contrast, overexpression of CMKLR1 by transfecting the plasmid encoding CMKLR1 markedly recovered the inhibition of LCN2 by CMKLR1 depletion (Figure 3F). Functionally, overexpression of LCN2 by plasmid significantly recovered the inhibition of VSMCs proliferation and migration resulting from CMKLR1 depletion (Figure 3H, 3I and Supplementary Figure 2). In agreement with this finding, overexpression of LCN2 also greatly recovered the expressions of PCNA and MMP-9 inhibited by CMKLR1 depletion (Figure 3G).

We then sought to explore the possible involvement of LCN2 in CMKLR1 depletion-induced inhibition of VSMCs proliferation and migration. As shown in Figure 3J, we determined a series of downstream signaling pathways of CMKLR1. Overexpression of LCN2 by plasmid significantly recovered the CMKLR1 depletion-induced inhibition of β-catenin, as well as p-p38, which is a key factor upregulating cellular migration. In agreement with this finding, knock down of LCN2 also greatly abolished the expressions of β-catenin and p-p38 triggered by CMKLR1 overexpression (Figure 3K). These results demonstrate the crucial role of LCN2 in upregulating CMKLR1-induced VSMCs proliferation and migration via the p38 / MAPK and Wnt / β-catenin signaling pathways.

### Curcumin plays a key role in regulating VSMCs proliferation and migration during AS by significantly inhibiting the expression of the chemerin / CMKLR1 / LCN2 axis

**Figure 4:**
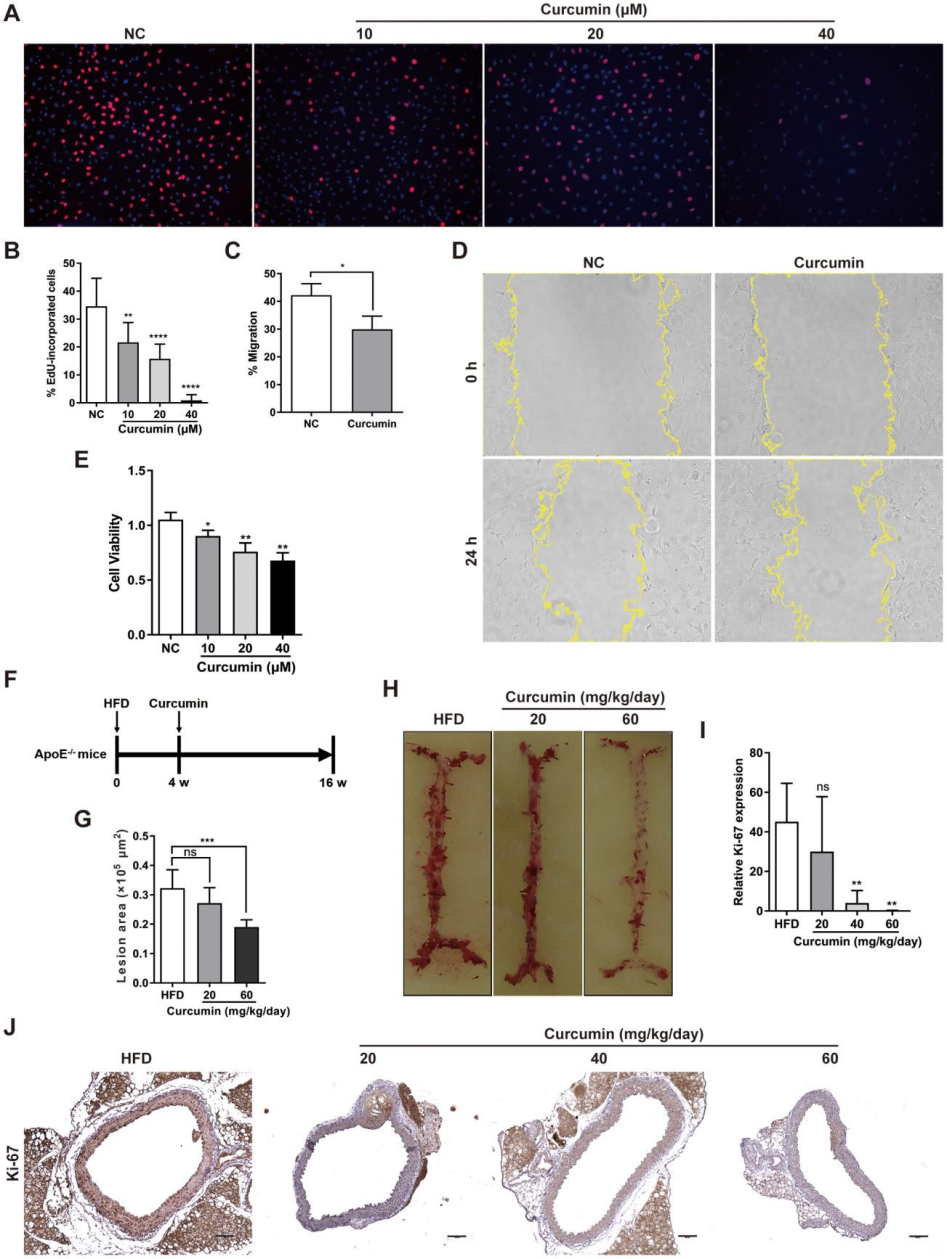
**Curcumin inhibits AS plaque by decrease VSMCs proliferation and migration.** (**A**, **B**) Analysis of percentage EdU-incorporated cells following curcumin treatment (48 h) for different durations. **, *p*< 0.01; ****, *p*< 0.0001. (**C**, **D**) Cell migration with or without curcumin treatment (20 μM, 24 h) as determined by wound healing assay. Percentage cell migration data are presented as means ± SDs. *, *p*< 0.05. (**E**) CCK-8 assessment of cell proliferation following treatment with curcumin (48 h) for different durations. *, *p*< 0.05; **, *p*< 0.01. (**F**) Schematic illustration of the establishment of a HFD ApoE^-/-^ mice model combined with curcumin treatment. HFD group, n = 8; curcumin (20 mg/kg/day), n = 8; curcumin (40 mg/kg/day), n = 8; curcumin (60 mg/kg/day), n = 8. (**G**, **H**) The lesion areas in aorta tissues of HFD ApoE^-/-^ mice with or without curcumin treatment. n.s., no significant; ***, *p*< 0.001. (**I**, **J**) IHC analysis of Ki-67 expression in aorta tissues of HFD ApoE^-/-^ mice with or without curcumin treatment. Scale bar = 100 μm. Red arrows indicate plaques. Ki-67 expression score analysis data are presented as means ± SDs. n.s., no significant; **, *p*< 0.01.

Curcumin has been demonstrated to inhibit early atherosclerotic lesions [21], but its role in regulation of CMKLR1-induced VSMCs proliferation and migration is unclear. Hence, we first examined the role of curcumin in VSMCs proliferation and migration *in vitro.* We demonstrated that curcumin inhibited VSMCs proliferation, migration (Figure 4A–4D) and cell viability (Figure 4E) in a dose dependent manner via the EdU incorporation and wound healing assays. To further explore the role of curcumin in regulating AS development, an HFD-induced AS model was used in combination with curcumin treatment (Figure 4F). As shown in Figure 4G, 4H, curcumin treatment significantly inhibited atherosclerotic plaque formation, and consistent with previous findings *in vitro,* we confirmed that the treatment also inhibited the aortic expression of Ki-67 in a dose dependent manner. These findings suggest that curcumin prevents AS progression by inhibiting VSMCs proliferation and migration.

**Figure 5:**
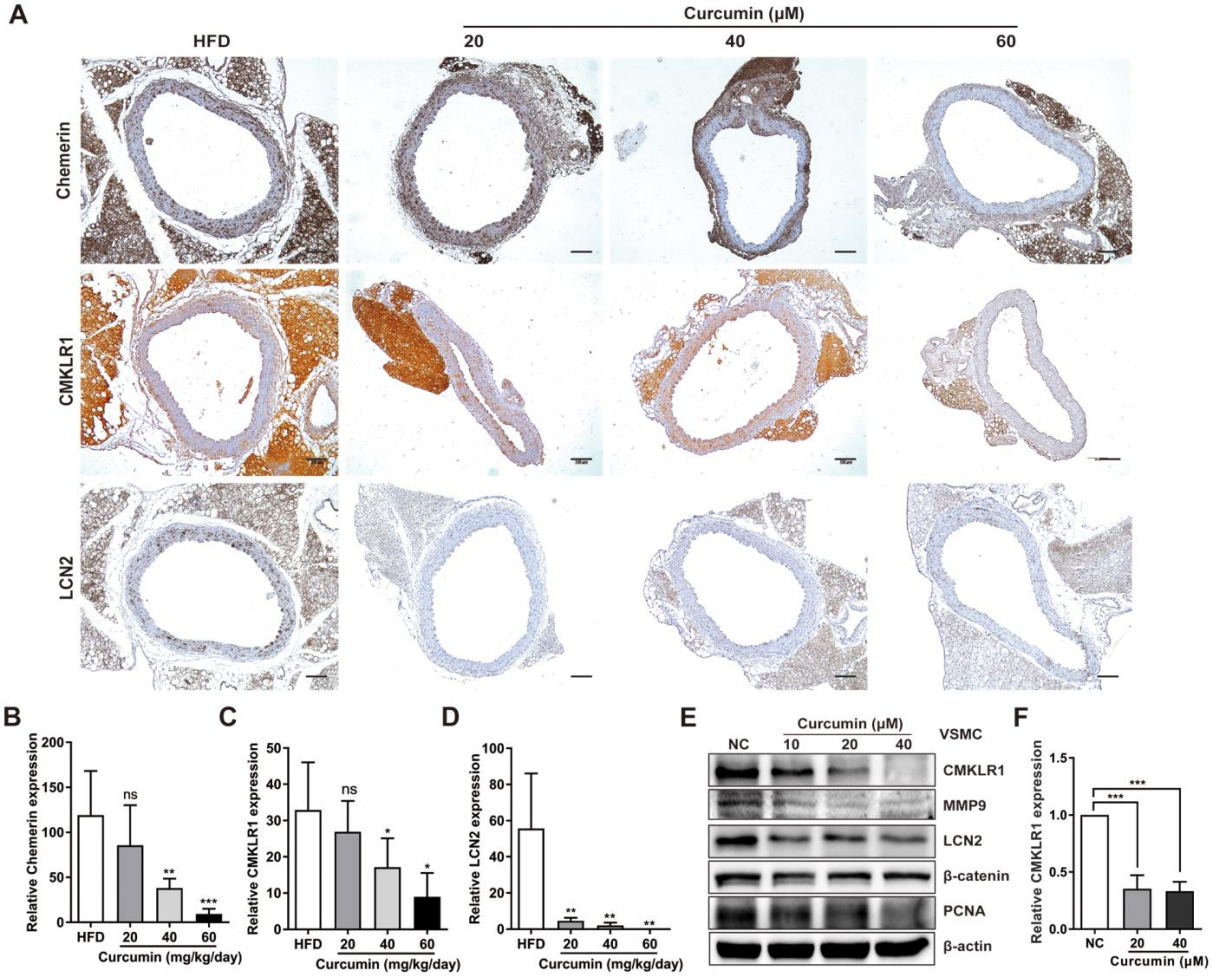
**Curcumin inhibits CMKLR1 and LCN2 expression.** (**A**) IHC analysis of chemerin / CMKLR1 and LCN2 expressions in aorta tissues of HFD ApoE^-/-^ mice with or without curcumin treatment. Scale bar = 100 μm. Red arrows indicate plaques. (**B**–**D**) Chemerin / CMKLR1 and LCN2 expression score analysis data are presented as means ± SDs. n.s., no significant; *, *p*< 0.05; **, *p*< 0.01; ***, *p*< 0.001. (**E**) Western blots showing CMKLR1, MMP-9, LCN2, β-catenin and PCNA expressions in cells treated with curcumin (48 h) for different durations. (**F**) mRNA expression of CMKLR1 with or without curcumin treatment (48 h) as determined by quantitative real-time PCR.

We then sought to explore the possible involvement of curcumin in regulating chemerin / CMKLR1 / LCN2 axis *in vitro*. We found that treatment with curcumin dramatically inhibited the expression of chemerin, CMKLR1, and LCN2 induced by HFD (Figure 5A–5D) in a dose dependent manner, and chemerin and CMKLR1 expression in the aorta, but not in the perivascular adipose tissue (Figure 5A, 5B). Hence, we confirmed that curcumin treatment significantly inhibited the protein expressions of chemerin / CMKLR1 / LCN2, as well as the mRNA expression of CMKLR1 (Figure 5E, 5F). Altogether, these results support the crucial role of curcumin in regulating VSMCs proliferation and migration by targeting the chemerin / CMKLR1 / LCN2 axis.

## DISCUSSION

In recent years, chemerin, an important adipokine, has gained increasing attention in relation to metabolic disease prevention and therapy. The chemerin / CMKLR1 signaling in regulating cellular proliferation and migration is a complex network [15]. By binding chemerin to CMKLR1, chemerin / CMKLR1 signaling plays crucial roles in mobilizing calcium, activating the PI3K and MAPK pathways, and recruiting β-arrestin 1 and β-arrestin 2. It is worth noting that the role of chemerin / CMKLR1 axis plays in development of AS remains contentious [10, 16]. In this study, an HFD mouse model was successfully established to investigate the role of chemerin / CMKLR1 in AS. At first, aorta expressions of chemerin and CMKLR1 were detected in mice with and without HFD. Then the primary VSMCs was cultured and treated with CMKLR1 agonist. Our results showed that the chemerin and CMKLR1 expressions in the aorta increased significantly in HFD mice compared to the control group. Importantly, CMKLR1 activation is able to promote the proliferation and migration of VSMCs *in vitro*. Our findings provide evidence that the elevated expression of chemerin and its receptor CMKLR1 might activate VSMCs proliferation and migration to induce AS.

Adipokines exert both independent and joint effects on cellular signal networks implicated in cellular proliferation, growth, migration and metastasis, thus it is necessary to explore the relationship between chemerin / CMKLR1 with other adipokines. LCN2 is dysregulated in multiple diseases like cancers in which it facilitates tumorigenesis by promoting survival, growth, and metastasis [28, 29]. Consistently with the discovery that LCN2 contributes to experimental AS in a stage-dependent manner [30], we determined that the aortic expression of LCN2 increased significantly in HFD mice compared to the control group. Interestingly, a recent study reported there exists a positive correlation between chemerin / CMKLR1 and LCN2 in human circulating levels [27]. In this study, we examined augmented chemerin / CMKLR1 expression with up-regulated LCN2 levels in the aorta of HFD mice, which was further confirmed by the fact that the mRNA and protein levels of LCN2 were positively regulated by CMKLR1. These results indicated that LCN2 may be involved in CMKLR1 signaling to control VSMCs proliferation and migration in AS, supported by our findings via EdU incorporation and wound healing assays. LCN2 is known to be a key regulator of cellular proliferation via Wnt / β-catenin signaling [26], which was also verified by our work showing that CMKLR1 or LCN2 knockdown remarkably downregulated β-catenin expression. We further confirmed that LCN2 upregulated CMKLR1-induced VSMCs proliferation and migration via the p38 / MAPK and Wnt / β-catenin signaling pathways. Crosstalk between the p38 / MAPK and Wnt / β-catenin signaling is an interesting issue. On one hand, p-p38 was reported to be necessary for suppressing the expression of Wnt signaling antagonists DKK1 and SFRP3, which inhibit the cellular proliferation [31]; on the other hand β-catenin depletion increased p38 MAPK phosphorylation via downregulation of PPARδ, whereas it had no effect on ERK phosphorylation. Functionally, the Wnt cascade was reported to upregulate the proliferation of VSMCs while the activation of p38 MAPK signaling can trigger VSMCs proliferation and migration [32, 33]. However, the specific influence of CMKLR1 on LCN2 expression warrants further investigation, which may provide more solid evidence on chemerin / CMKLR1 / LCN2-induced AS. One possible mechanism is that CMKLR1 activation may upregulate LCN2 expression by promoting the Akt-c-Myc pathway.

The role of curcumin in AS has been widely reported and the mechanisms include regulation of cholesterol metabolism, low-density lipoprotein oxidation, and inhibition of inflammation [34–36]. Consistent with previous studies [21], we discovered that curcumin treatment significantly inhibited the formation of atherosclerotic plaques in HFD-induced AS mice. Meanwhile, we demonstrated that curcumin could inhibit aortic proliferation using Ki-67 staining. Further, we demonstrated that curcumin significantly inhibited VSMCs proliferation and migration in a dose-dependent manner *in vitro*. Therefore, we clearly discovered curcumin inhibits VSMCs proliferation in our HFD-induced AS mice. More importantly, we observed that curcumin significantly reduced chemerin / CMKLR1 expression in the aorta of HFD-induced AS mice in a tissue-specific manner. And we also found curcumin inhibits the chemerin / CMKLR1 / LCN2 signaling pathway *in vivo* and *in vitro.* Collectively, it is very probable for curcumin to inhibit VSMCs proliferation by targeting CMKLR1 / LCN2 signaling, although the specific mechanisms are still awaiting to be further explored.

In conclusion, our study demonstrated the positive role of the chemerin / CMKLR1 signaling pathway in VSMCs proliferation and migration during as. Importantly, we discovered that LCN2 is downstream target of the chemerin / CMKLR1 signaling pathway and is required for its upregulation of VSMCs proliferation and migration via the p38 / MAPK and Wnt / β-catenin signaling. We also confirmed that curcumin inhibits VSMCs proliferation and migration by inhibiting chemerin / CMKLR1 / LCN2, thereby inhibiting AS progression. This suggests that the chemerin / CMKLR1 / LCN2 signaling pathway is a potential target for AS clinical therapy.

## MATERIALS AND METHODS

### Cell culture and reagents

For the isolation and culture of primary VSMCs, eight-week-old C57BL/6 mice were anesthetized and soaked in 75% ethanol. The chest of each mouse was opened, and the aorta was completely separated then placed in 35-mm wells containing phosphate-buffered saline (PBS) or Dulbecco’s Modified Eagle’s Medium (DMEM). The outer membrane of the blood vessel was torn, and the blood vessel was cut into 1 mm^2^ tissue pieces with ophthalmic scissors. The tissue pieces were attached to the 35-mm dish and incubated upside down at 37° C under a humidified atmosphere with 5% CO_2_ for 30 min. Then, fresh complete culture medium was added after the tissue attachment to the dish. Primary VSMCs grown from the tissue were used for all experiment between 4-10 passages.

Curcumin (Sigma-Aldrich, C1386, USA) was diluted in dimethyl sulfoxide (DMSO, 2650, Sigma-Aldrich, USA); the stock solution was 50 mM. Dilute solutions of all reagents were used within 1 month. Recombinant mouse chemerin-9 (Catalog number: 2325-CM-025) was purchased from R&D systems, USA, and the stock solution was dissolved in PBS containing at least 0.1% 100 μg/ml bovine serum albumin (BSA).

### Animal model

Male ApoE-/- mice (6-8 weeks old) obtained from Gempharmatech of Information Technology Center (Nanjing, China) were used for the AS model, and all animal experiments were maintained in a specific pathogen-free laboratory animal center of Shenzhen People’s Hospital. All animal maintenance and procedures were carried out in strict accordance with the recommendations established by the Animal Care and Ethics Committee of Shenzhen People’s Hospital and the Guidelines for Animal Experiments set by the Bureau of Sciences and Techniques of Guangdong Province, China (Research Ethics Board approval number: SYXK2007-0025).

Negative control (NC) group ApoE-/- mice were fed with normal diet. HFD group mice were fed with 60% kcal high-fat diets (BIOPICK, D12492, China). Curcumin group mice were fed with 20 40, 60 or 100 mg/kg/day by gavage. NC and HFD group mice were fed for 16 weeks before sacrifice. For the curcumin group, ApoE-/- mice were fed with HFD for 1 month and then with different doses of curcumin and HFD for another 3 months.

### Immunohistochemistry (IHC) staining

Artery specimens (4 μm slices) were harvested for immunohistochemical staining, dewaxed, rehydrated with different alcohol concentrations. Slides were incubated with 5% goat serum at room temperature for 30 min, and antibodies with optimal concentrations used for the study proteins were incubated overnight. Freshly prepared 33’-diaminobenzidine substrate solution was added to each section. Slides were then differentiated with 1% hydrochloric acid alcohol, dehydrated and restored to blue color with ammonia at different concentrations (from low to high), and further dehydrated until transparent in xylene. Then the slides were sealed with neutral gum.

### EdU (5-Ethynyl-2'-deoxyuridine) incorporation assay

VSMCs were plated in a 6-well plate at a 1 × 10^5^ /well density. Then, 1× EdU solution was incubated for 5 h and 1 ml fixed fluid was added for 15 min after removing EdU solution and 1 ml transparent liquid was added for 10 min. Cells were incubated with 500 μl Click Additive Solution at room temperature in the dark for 30 min, washed thrice with wash buffer and the optical density value at 495 nm was measured (Beyoclick^TM^ EdU cell proliferation kit with Alexa Fluor 555; Beyotime, 0075S, China).

### Wound healing assay

VSMCs were cultured in 6-well cell plates with complete medium until 100% confluent. A 200 μl pipette tip scratched the surface of the cells and the cells were washed twice with PBS. Then, pictures of each “wound” were taken using an inverted microscope (Olympus, Tokyo, Japan). Cells were incubated at 37° C containing 5% CO_2_ and pictures of each time point and concentration were taken microscopically.

### Cell counting kit-8 (CCK8)

Cell viability was determined using a CCK-8 kit (DoJinDo, CK04, Japan) according to the manufacturer’s instructions. VSMCs (0.5 × 10^4^ cells/well) were seeded into a 96-well plate before treatment and cultivated for 48 h with curcumin or chemerin-9. Then, cells were treated with 10 μl CCK8 reagent diluted in 90 μl DMEM medium and incubated for 2 h at 37° C with 5% CO_2_ incubator. The density was measured using a Spark 10M multimode microplate reader (Tecan, Switzerland) at 450 nm. Three replicates were prepared for each concentration.

### Lentiviral infection

Lentiviruses expressing shRNA targeting SC or CMKLR1 were purchased from GenePharma (Shanghai, China). For the lentiviral infection, VSMCs were cultured with viral supernatant (multiplicity of infection, MOI = 100) for 24 h and were then selected with 2 μg/ml of puromycin for 3 days. Puromycin (1 μg/ml) was used to maintain stable cells. The targeting sequences of shCMKLR1 are specified in Supplementary Table 1.

### Western blot

VSMCs were lysed in RIPA buffer (Beyotime, P0013, China) containing 1 mM phenylmethylsulphonyl fluoride (Beyotime Institute of Biotechnology, Haimen, China) and 1 mM phosphatase (protease inhibitor cocktail tablets; Roche, Mannheim, Germany) for extracting whole cell protein samples. Protein concentration was detected using a bicinchoninic acid protein assay kit (Beyotime, P0012, China). Protein samples (20-40 μg) were separated on SDS-PAGE gels and then transferred onto polyvinylidene difluoride membranes (ISEQ00010, Merck Millipore Ltd, Germany) for 1 h. Protein band intensities were evaluated using an electrochemiluminescent (ECL) western blotting kit (Merck Millipore Ltd, WBKCS0500, Billerica, Germany) and normalized to beta-actin. All experiments were performed at least three times. β-actin (Abcam, ab6276, U.S.A), chemerin (Abcam, ab203040, U.S.A), CMKLR1 (Invitrogen, PA5-50932, U.S.A), PCNA (Cell Signaling Technology, 13110, U.S.A), β-catenin (Abcam, ab2365, U.S.A) and MMP9 (Abcam, ab58803, U.S.A) antibodies were used.

### Reverse transcription and real-time PCR

Total RNA was extracted from VSMCs or tissues using TRIZOL reagent (Invitrogen, 15596026, CA, USA). Based on the instructions on the reverse transcriptase kit (Primescript^TM^ RT reagent kit with gDNA Eraser; Perfect Real Time; Takara, RR047A, Tokyo, Japan), cDNA was synthesized using 1 μg of the total RNA. cDNA samples were diluted and subjected to qRT-PCR for 40 cycles using TB Green TM Premix Ex Taq^TM^ II (Takara, RR820A, Tokyo, Japan). Primers (Supplementary Table 2) were synthesized at Sango Biotech Co. Ltd. (Shanghai, China). β-actin (B661302, Sangon biotech, Shanghai, China) was used as an internal control. Data of different genes were analyzed using the comparative Ct (2^-ΔΔCT^) method.

### Small interfering RNA (siRNA) transfection

siRNA targeting LCN2 sequence was used for knockdown experiments (GenePharma, Shanghai, China). Cells were cultured overnight at a density of 1 × 10^5^ cells in a 6-well plate. siRNA targeting LCN2 was transfected into cells using RNAimax (Lipofectamine™ RNAiMAX Transfection Reagent; Invitrogen, 13778150, CA, USA) for 8 h. Cells were then cultured with fresh complete medium for 3 days. Targeting sequence of the above siRNA is specified in Supplementary information (Supplementary Table 3).

### Oil red staining

The ApoE-/- mice were exposed to the heart, and the origin of the autonomous artery was dissected and separated from the whole aorta to the bifurcation of the common iliac artery of the abdominal aorta. The dissected aorta was washed with normal saline and placed in the fixator (4% paraformaldehyde, 5% sucrose, 2 μmol/l ethylenediaminetetraacetic acid, pH 7.4). It was then stained with 0.5% Sudan IV staining solution (0.5 g Sudan IV, 50 ml acetone, 50 ml 70% ethanol) for 6 min, and transferred to 80% ethanol for decolorization. Pictures were taken using a Digital Single Lens Reflex Camera (Canon, Japan).

### Statistical analysis

All experiments were performed three times, and the data were expressed as means ± SD. Statistical analysis was performed using GraphPad Prism 6 (GraphPad Software, San Diego, CA, USA). Comparison between two groups was conducted using the Student's t-test; *p*< 0.05 was considered statistically significant.

### Transparency document

The transparency document associated with this article can be found in the online version.

## Supplementary Material

Supplementary Figures

Supplementary Tables
