# Supplementary material for: Curcumin inhibits the proliferation and migration of vascular smooth muscle cells by targeting the chemerin / CMKLR1 / LCN2 axis
Source: Aging (Albany NY). 2021 May 24;13(10):13859–75. doi: 10.18632/aging.202980 (PMC8202847; doi:10.18632/aging.202980)
Supplement: Supplementary Figures [file aging-13-202980-s001.pdf]

SUPPLEMENTARY FIGURES

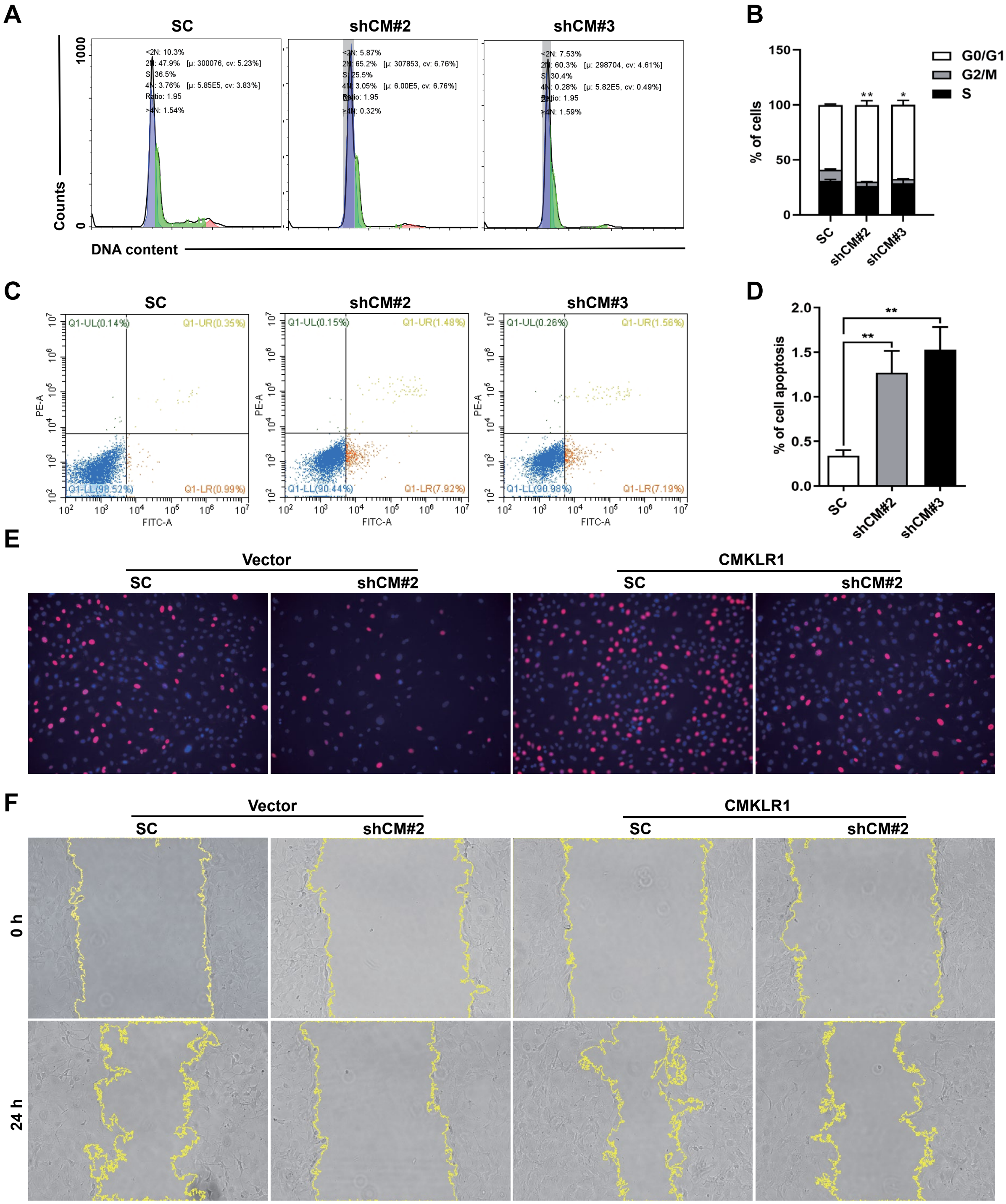

Supplementary Figure 1. Downregulation of CMKLR1 inhibit VSMCs proliferation and migration. (**A**) CMKLR1 depletion induced VSMCs cell cycle arrest, as assessed by flow cytometry analysis. **, p< 0.01; *, p< 0.05. (**B**) Percentage of apoptotic cells as determined by flow cytometry and analyzed using Flowjo software. **, p< 0.01. (**C**) Analysis of the EdU-incorporated cells following the application of plasmids encoding CMKLR1 to overexpress CMKLR1 expression in VSMCs with or without CMKLR1 knockdown. (**D**) Cell migration as determined by wound healing assay. (**E**) Analysis of the EdU-incorporated cells following the application of plasmids to overexpress CMKLR1 expression in VSMCs with or without CMKLR1 knockdown. (**F**) Cell migration as determined by wound healing assay.

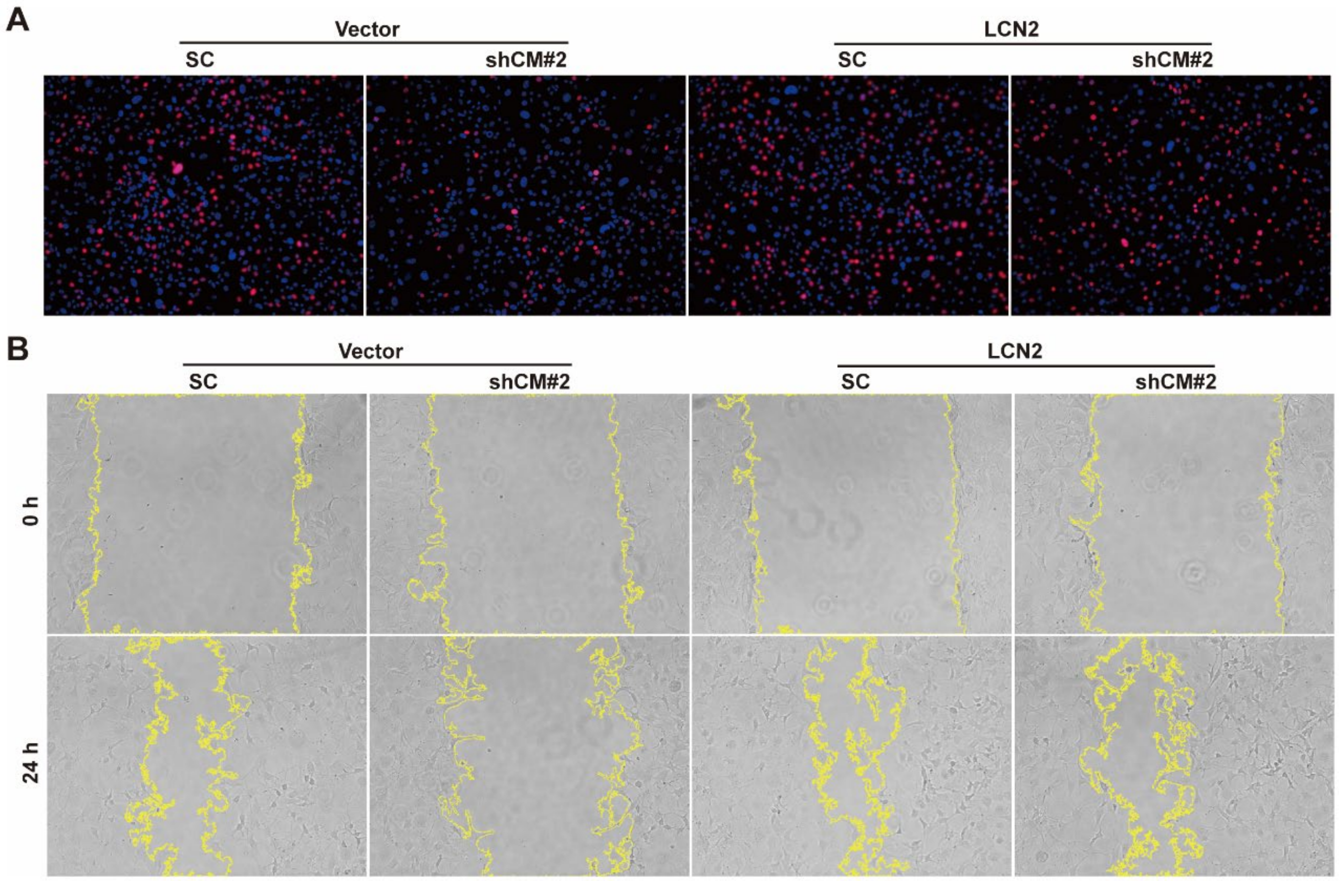

Supplementary Figure 2. LCN2 is essential for the inhibition of VSMCs proliferation by CMKLR1 depletion. (**A**) Analysis of the EdU-incorporated cells following the application of plasmids encoding LCN2 to overexpress LCN2 expression in VSMCs with or without CMKLR1 knockdown. (**B**) Cell migration as determined by wound healing assay.
