# Supplementary material for: Curcumin inhibits the proliferation and migration of vascular smooth muscle cells by targeting the chemerin / CMKLR1 / LCN2 axis
Source: Aging (Albany NY). 2021 May 24;13(10):13859–75. doi: 10.18632/aging.202980 (PMC8202847; doi:10.18632/aging.202980)
Supplement: Supplementary Tables [file aging-13-202980-s002.pdf]

SUPPLEMENTARY TABLES

Sequences using for shRNA, the real-time PCR, siRNAs

Supplementary Table 1. Sequences of shRNAs for CMKLR1.

Target	Sequence
shCMKLR1#2	GGAAGATAACCTGCTTCAACA
shCMKLR1#3	GCTACCTTACCATCGTCTTCA
Scramble control	TTCTCCGAACGTGTCACGT

Supplementary Table 2. List of the primers used for the real-time PCR.

Target gene	Forward	Reverse
LCN2	GGCTGTCGCTACTGGATCAGAAC	CGAACTGGTTGTAGTCCGTGGTG
CMKLR1	GTACACCAGCGTCTTCCTGC	TCCCATGAATGTTGGCGGTG

Supplementary Table 3. List of the siRNA sequences targeting LCN2.

Target gene	Forward	Reverse
LCN2	GAUCAGAACAUUUGUUCCATT	UGGAACAAAUGUUCUGAUCTT
